# Molecular and Functional Characterization of Grapevine NIPs through Heterologous Expression in *aqy-*Null *Saccharomyces cerevisiae*

**DOI:** 10.3390/ijms21020663

**Published:** 2020-01-19

**Authors:** Farzana Sabir, Sara Gomes, Maria C. Loureiro-Dias, Graça Soveral, Catarina Prista

**Affiliations:** 1 LEAF, Linking Landscape, Environment, Agriculture and Food, and DRAT, Dept. de Recursos Biológicos, Ambiente e Território, Instituto Superior de Agronomia, Universidade de Lisboa, Tapada da Ajuda, 1349-017 Lisboa, Portugal; mcdias@isa.ulisboa.pt (M.C.L.-D.); cprista@isa.ulisboa.pt (C.P.); 2 Research Institute for Medicines (iMed.ULisboa), Faculty of Pharmacy, Universidade de Lisboa, 1649-003 Lisboa, Portugal; gsoveral@ff.ulisboa.pt; 3 IBB, Institute of Bioengineering and Biosciences, Instituto Superior Técnico, Universidade de Lisboa, 1049-001 Lisboa, Portugal; saracgomes@tecnico.ulisboa.pt

**Keywords:** aquaporin, grapevine, *Saccharomyces cerevisiae*, NIPs, metalloids, glycerol

## Abstract

Plant Nodulin 26-like Intrinsic Proteins (NIPs) are multifunctional membrane channels of the Major Intrinsic Protein (MIP) family. Unlike other homologs, they have low intrinsic water permeability. NIPs possess diverse substrate selectivity, ranging from water to glycerol and to other small solutes, depending on the group-specific amino acid composition at aromatic/Arg (ar/R) constriction. We cloned three *NIPs* (*NIP1;1*, *NIP5;1*, and *NIP6;1*) from grapevine (cv. Touriga Nacional). Their expression in the membrane of *aqy-null Saccharomyces cerevisiae* enabled their functional characterization for water and glycerol transport through stopped-flow spectroscopy. *Vv*TnNIP1;1 demonstrated high water as well as glycerol permeability, whereas *Vv*TnNIP6;1 was impermeable to water but presented high glycerol permeability. Their transport activities were declined by cytosolic acidification, implying that internal-pH can regulate NIPs gating. Furthermore, an extension of C-terminal in *Vv*TnNIP6;1M homolog, led to improved channel activity, suggesting that NIPs gating is putatively regulated by C-terminal. Yeast growth assays in the presence of diverse substrates suggest that the transmembrane flux of metalloids (As, B, and Se) and the heavy metal (Cd) are facilitated through grapevine NIPs. This is the first molecular and functional characterization of grapevine NIPs, providing crucial insights into understanding their role for uptake and translocation of small solutes, and extrusion of toxic compounds in grapevine.

## 1. Introduction

The sessile nature of plants restricts their access to water and essential minerals in their vicinity. To overcome this limitation, membrane channel diversity has been suggested as a sophisticated evolvement in plants [1,2]. Plant genomes have several-fold more Major Intrinsic Protein (MIP) genes than other organisms, implying their multiple functions for plant growth and adaptation, especially under stress [1]. Aquaporins, the MIP family members, are crucial for water and ion homeostasis in all living forms [3]. Up/down regulation of aquaporins has been shown under various stresses like drought and salinity, and nutrient deficiency/toxicity, suggesting their unique requirement under stress [1]. Plant aquaporins are divided into four major groups: (1) Plasma membrane Intrinsic Proteins (PIPs), (2) Tonoplast Intrinsic Proteins (TIPs), (3) Small basic Intrinsic Proteins (SIPs), and (4) Nodulin 26-like Intrinsic Proteins (NIPs) [1].

NIPs represent a unique subfamily of plant aquaporins because of their broad-spectrum substrates selectivity. The first identified member of the NIP group was Nod-26, localized in the nodules of *Glycine max*, where this archetype is involved in the exchange of water and metabolites between the host and the symbiont [4]. Nevertheless, the widespread occurrence of NIPs in non-leguminous plants, and their spatiotemporal expression during plant development suggest their broader function in water and nutrient homeostasis rather than being just restricted to the symbiotic function of Nod26 [5]. They facilitate the conductance of a wide range of beneficial solutes such as boron (B), selenium (Se), and silicon (Si), and are also considered crucial for extrusion of toxic minerals like arsenium (As), germanium (Ge), and antimony (Sb) (Reviewed by [6]). Additionally, NIPs are regarded as equivalent to the microbial aquaglyceroporins because they are the only membrane channels that transport glycerol in plants [5,7]. Extensive phylogenic studies of MIPs suggest that glycerol conductance in plants was acquired by a horizontal gene transfer (HGT) and functional recruitment of bacterial aquaporins (AQPs) [7,8]. The broad but unique substrate selection of NIPs is associated with numerous functions in plant development and adaptation to their continuously changing environment [6]. The two highly conserved Asn-Pro-Ala (NPA) motifs configure the aqueous pore for water transport, whereas the selection of other substrates is determined by the aromatic/Arg (ar/R) constriction formed at the extracellular end of the pore [9]. Based on the sequence similarities and amino acid composition in the ar/R region, NIPs are divided into three groups NIP-I, NIP-II, and NIP-III [10], possessing distinct estimated pore sizes, which allows the selection of diverse substrates. For instance, water-selective channels form more hydrophilic and smaller pore (diameter ~2.8 Å), whereas glycerol-transporting channels have a less-constricted pore (diameter ~3.4 Å) with relatively more hydrophobic residues [11,12].

*Vitis vinifera* is one of the most important economic fruit crops cultivated worldwide and holds the first position of the fully sequenced ligneous plant [13]. In vineyards, drought, salinity, and nutrient imbalance are major limitations [14]. In grapevine, 28 identified aquaporins genes [13] were clustered in four major subfamilies PIPs, TIPs, SIPs, and NIPs [15] together with the recently studied Uncharacterized Intrinsic Protein (XIP) [16]. A strong correlation between aquaporin expression and plant adaptation to abiotic stress has been established in grapevine [17]. Although little is known about the physiological relevance of NIPs in grapevine, they represent a unique molecular entry point for crucial solutes in stress responses. Their lower and specialized expression in plants and undefined regulation obstructs their functional interpretation. To address this problem, a heterologous expression system of *Saccharomyces cerevisiae* represents an important tool for characterizing grapevine aquaporins [15,16,18].

Comprehensive studies on grapevine NIPs will offer unique insights into the various physiological processes of plant life, especially substrate transport under nutrient deficiency/toxicity and phytoremediation of toxic substrates. In the present study, we cloned and heterologously expressed the grapevine NIPs in *aqy-null S. cerevisiae*. Their functional characterization for water and glycerol transport was performed by stopped-flow spectroscopy. Furthermore, the possibility of metalloid and other small solutes conductance was also examined by sensitivity/tolerance growth assays of the NIPs expressing yeast strains.

This study will contribute to the understanding of the NIPs role in the uptake and translocation of water/glycerol as well as micronutrients/metalloids substrates, and the extrusion of toxic compounds. The findings may provide the first crucial insight into the NIPs contribution toward water and mineral homeostasis and the phytoremediation process in grapevine.

## 2. Results and Discussion

### 2.1. Sequence Analysis and Expression of Grapevine NIPs in S. cerevisiae

The obtained NIP gene sequences from Touriga Nacional presented obvious similarity with the database sequences of Pinot Noir variety, except *NIP6;1* sequence. In this homolog, insertion of four base pairs (^927^TTCA) was found in Touriga Nacional, causing a premature stop codon, which eventually resulted in a shorter C-terminal protein (312aa) as compared to the database protein (354aa) (Figure S1). N- and C-terminals of NIP proteins were shown to be crucial for the gating, and their manipulation showed proper functional expression of NIPs in yeast [5,19]. To corroborate this notion, we genetically engineered the C-terminal of *Vv*TnNIP6;1. PCR-based site-directed mutagenesis was performed by using the primers listed in Table S1, to remove the four base pairs insertion, resulting in *Vv*TnNIP6;1M homolog with extended C-terminal. The mutated (*Vv*TnNIP6;1M) aquaporin had a similar length to *Vv*PnNIP6;1 of Pinot Noir (Figure S2). 

**Figure 1:**
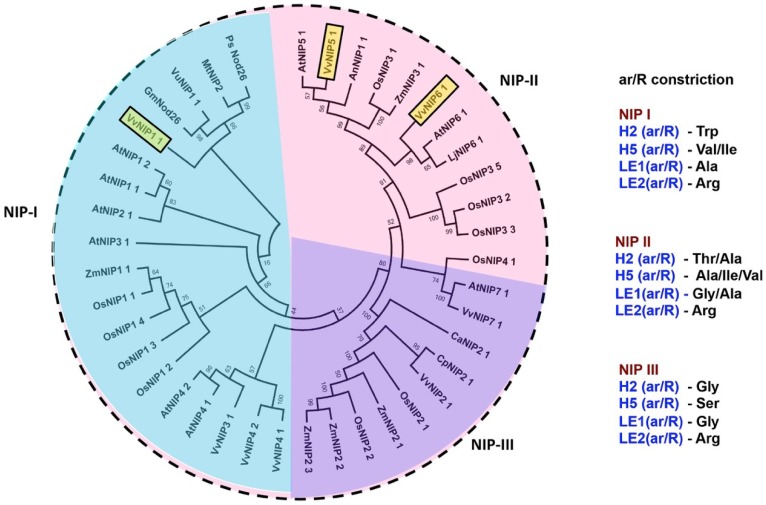
Phylogenetic tree of Nodulin 26-like Intrinsic Proteins (NIPs) aquaporins from various plant species. The tree was constructed by MEGA7.0 software using neighbor-joining method with 1000 bootstrap replicates [22]. Grapevine NIPs, obtained in the present study, are boxed. The division between three groups of NIPs (NIP-I, NIP-II, and NIP-III) is indicated in different colors. Amino acid compositions at aromatic/Arg (ar/R) constriction of each NIP group are mentioned in the figure. To identify the origin of each NIP, a species acronym is used as the prefix of the protein; An: *Atriplex nummularia*; At: *Arabidopsis thaliana*; Ca: *Cicer arietinum*; Cp: *Cucurbita pepo*; Cs: *Cucumis sativus*; Gm: *Glycine max*; Lj: *Lotus japonicus*; Mt: *Medicago truncatula*; Os: *Oryza sativa*; Pit: *Pinus taeda*; Pp: *Physcomitrella patens*; Pt: *Populus trichocarpa*; Sb: *Sorghum bicolor*; Vu: *Vigna unguiculata*; *Vv*: *Vitis vinifera*; Zm: *Zea* *mays*.

Based on sequence similarity and conserved MIP domains, the whole-genome sequencing of *Vitis vinifera* revealed the existence of a total of eight *NIP* genes [13]. More than forty NIP sequences of various plant sources were collected and aligned to construct the phylogenetic tree. Figure 1 demonstrates that all NIPs, including from grapevine, are distinctly distributed in three groups. Four out of eight grapevine NIPs (NIP1;1, NIP3;1, NIP4;1, and NIP 4;2) were grouped in the NIP-I group. *Vv*NIP1;1 was closely clustered with archetype *Gm*Nod26, whereas *Vv*NIP3;1, *Vv*NIP4;1, and *Vv*NIP4;2 were grouped with *At*NIP4;1 and *At*NIP4;2. The NIP-II group comprised the grapevine *Vv*NIP5;1 and *Vv*NIP6;1, the former one was closely grouped with *At*NIP5;1, while the later one was clustered with *Arabidopsis* and *Lotus* NIP6;1. Whereas *Vv*NIP7;1 and *Vv*NIP2;1 belong to NIP-III group.

Sequence analysis of ar/R constriction of each group supports the hypothesis of NIPs classification basis [10]. The amino acid residues in the ar/R region of NIP-I (W-V/I-A-R), NIP-II (T/A-A/I/V-G/A-R), and NIP-III (G-S-G-R) are exclusively group-specific (Figure 1). The ar/R constriction determines the hydrophobicity/philicity and size of the pore, which eventually defines the size and nature of the substrates [20]. Numerous studies of NIPs molecular modeling and phylogenetic analysis have hypothesized that members of the NIP-I group can transport water as well as glycerol, while the NIP-II group comprised members with wider pore and more diverse substrate selectivity. The third cluster of NIP-III group contains hydrophilic residues and widely open ar/R filter, permeable to more bulky substrates [5,10,21].

**Figure 2:**
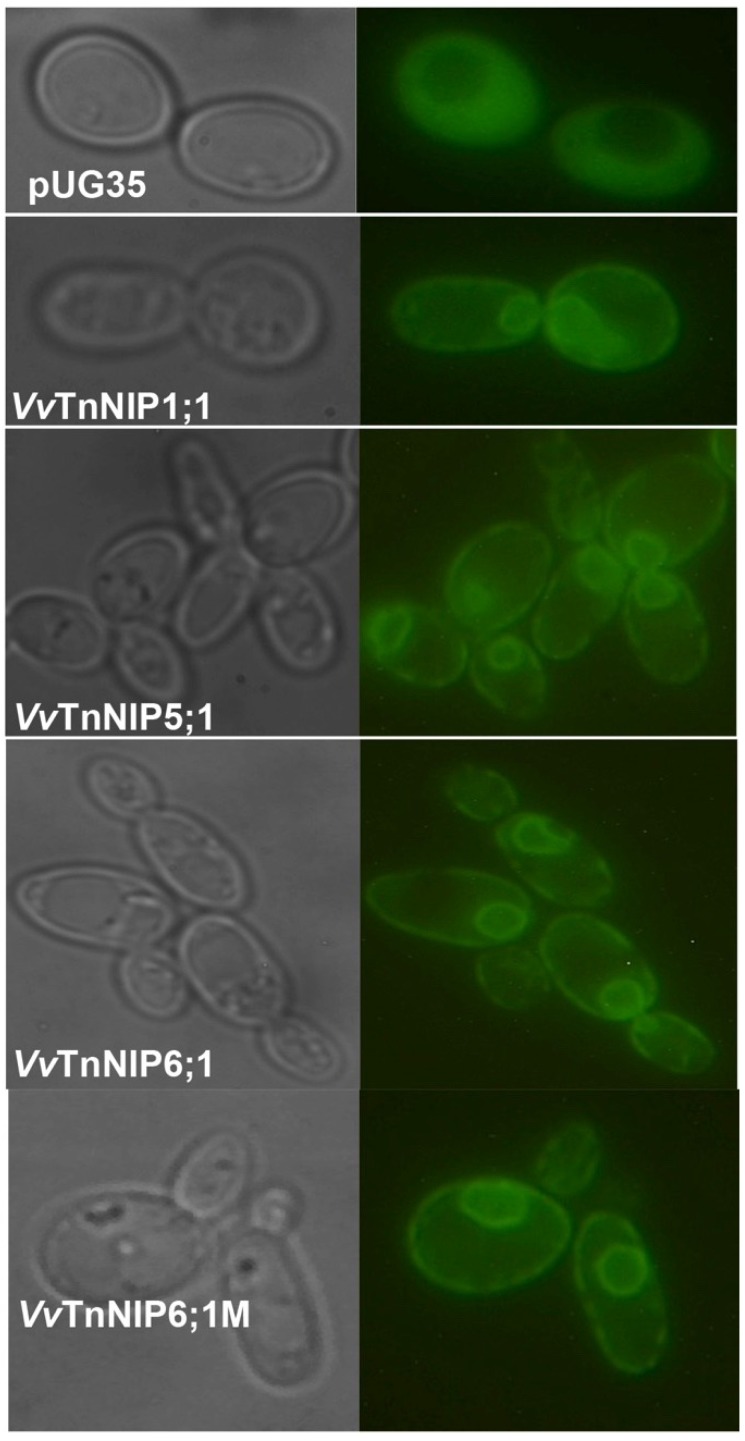
Membrane localization of GFP-tagged grapevine NIPs in *aqy-null S. cerevisiae*. Yeast cells transformed either with pUG35-GFP (control) or pUG35-*Vv*TnNIPs-GFP were observed under phase contrast (left panel) and fluorescence microscopy (right panel) at 100× magnification.

Fluorescent microscopy of *S. cerevisiae* strains revealed that the majority of the expressed GFP-tagged grapevine NIPs fragment was localized in the yeast membrane. Whereas, some fraction was retained at intracellular structures (Figure 2), similarly to our previous study of PIPs and TIPs, suggesting their retention in the endoplasmic reticulum or secretory route vesicles of the yeast expression system [15].

### 2.2. Water and Glycerol Transport Assays by Stopped-Flow Spectroscopy

**Figure 3:**
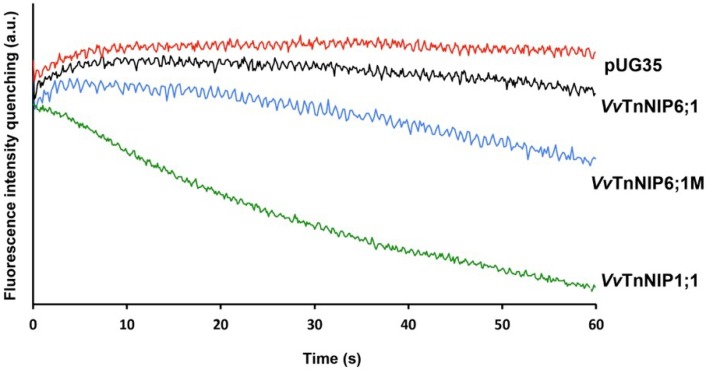
Traces obtained from stopped-flow spectroscopy. The hyperosmotic shock (with 2.1 M glycerol) was applied to the yeast cells (equilibrated with 1.4 M sorbitol) expressing functional NIPs (*Vv*TnNIP1;1, *Vv*TnNIP6;1) of grapevine and empty plasmid pUG35. C-terminal extension in *Vv*TnNIP6;1M homolog showed higher glycerol influx rate, whereas, *Vv*TnNIP1;1 strain exhibited highest glycerol influx.

Yeast cells expressing grapevine NIPs were assayed for water and glycerol transport activity by stopped-flow spectroscopy. Figure 3 shows typical fluorescence signals produced by changes in cell volumes when yeasts are challenged with glycerol osmotic gradients, with consequent glycerol influx, presumably, through grapevine NIPs.

**Figure 4:**
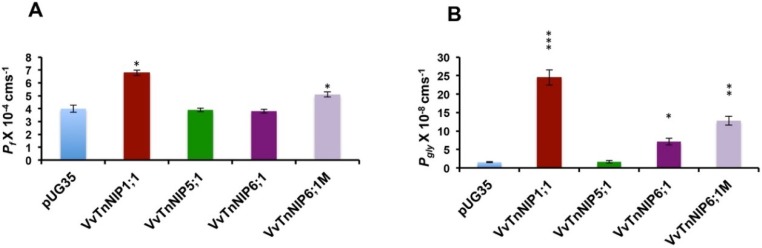
Water (*P_f_*) and glycerol (*P_gly_*) permeability coefficients measurement. The permeabilities were measured in yeast strains expressing grapevine NIPs and empty vector pUG35 at 23 °C and pH 5.0. (**A**) Expression of *Vv*TnNIP1;1 resulted in 1.7-fold enhanced water permeability, whereas the other NIPs could not increase the water permeability. C-terminal extension in *Vv*TnNIP6;1M homolog slightly enhanced the water permeability. (**B**) A 15-fold improved glycerol permeability was observed due to *Vv*TnNIP1;1 expression. Whereas, in *Vv*TnNIP6;1M strain, a 1.8-fold increased glycerol permeability was observed as compared to native *Vv*TnNIP6;1 expressing strain. Data is represented in the mean ± SD of three independent experiments with ten traces. Statistically significant differences are shown as an asterisk, calculated by *t*-test (* *p* < 0.05, ** *p* < 0.01, and *** *p* < 0.001).

**Figure 5:**
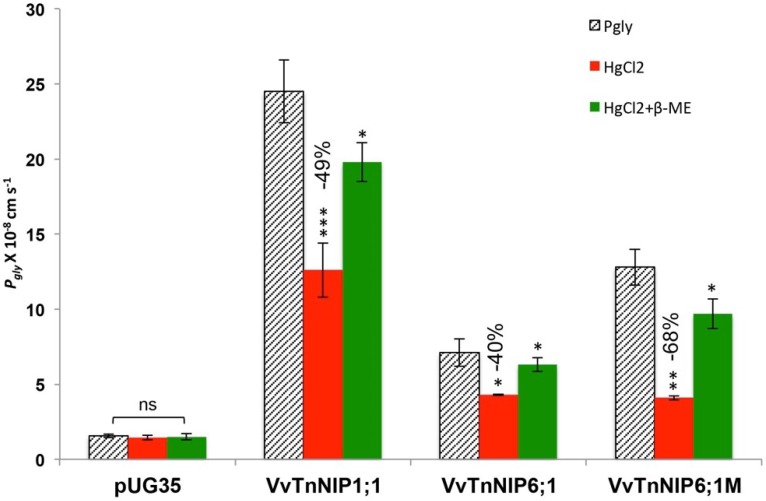
Inhibition of glycerol permeability (*P_gly_*) of grapevine NIPs by mercury chloride. Yeast strains expressing functional grapevine NIPs (*Vv*TnNIP1;1, *Vv*TnNIP6;1, *Vv*TnNIP6;1M) showed 40–68% reduction in glycerol permeability in the presence of 0.5 mM mercury chloride. The reversal of the inhibitory effect was observed when the cells were further incubated with 3 mM β-mercaptoethanol. Data is represented in the mean ± SD of three independent experiments with five traces. Statistically significant differences are shown as an asterisk, calculated by *t*-test (* *p* < 0.05, ** *p* < 0.01, and *** *p* < 0.001).

A significantly enhanced channel activity of *Vv*TnNIP1;1 for osmotic water (*P_f_*: 6.78 ± 0.21 × 10^−4^ cm s^−1^) and glycerol (*P_gly_*: 24.5 ± 2.1 × 10^−8^ cm s^−1^) transport was observed as compared to the empty plasmid expressing strain (*P_f_*: 4.0 ± 0.28 × 10^−4^ cm s^−1^ and *P_gly_*: 1.55 ± 0.11 × 10^−8^ cm s^−1^) (Figure 4). The increase in the permeabilities was consistent with a decrease in the activation energies for water (9.8 ± 0.15 kcal mole^−1^) and glycerol (6.93 ± 0.22 kcal mol^−1^) (Table 1). On the other hand, the expression of native *Vv*TnNIP6;1 homolog did not enhance the water permeability (3.8 ± 0.15 × 10^−4^ cm s^−1^), but mutated *Vv*TnNIP6;1M homolog slightly improved the water transport activity (*P_f_*: 5.1 ± 0.2 × 10^−4^ cm s^−1^ and *E_a_*: 11.3 ± 0.4 kcal mol^−1^) (Figure 4, Table 1). Similarly, a significantly improved glycerol transport activity was observed due to the expression of mutated *Vv*TnNIP6;1M homolog with longer C-terminal (*P_gly_*: 12.8 ± 1.2 × 10^−8^ cm s^−1^, *E_a_*: 8.6 ± 0.5 kcal mol^−1^) as compared to the native one (*P_gly_*: 7.1 ± 0.91 × 10^−8^ cm s^−1^, *E_a_*: 12.55 ± 0.8 kcal mol^−1^) (Figure 4, Table 1). Expression of *Vv*TnNIP5;1 did not improve the water and glycerol permeabilities. Glycerol permeabilities of functional *Vv*NIP channels were reduced in the presence of mercury chloride (Figure 5). A stronger inhibitory effect (68%) in mutated *Vv*TnNIP6;1M was observed on NIP mediated glycerol transport. Further on, the glycerol permeabilities were regained in the presence of mercaptoethanol, to approximately 80%, 89%, and 76%, in *Vv*TnNIP1;1, *Vv*TnNIP6;1, and *Vv*TnNIP6;1M expressing strains, respectively (Figure 5). 

The NIP channels have been demonstrated as microbial aquaglyceroporins, facilitating water as well as glycerol flux in plants [5]. However, this MIP subfamily presented lower intrinsic water permeability than the other subfamilies, PIPs, and TIPs [23]. In the present finding, the increase in water permeability (70% maximum) due to *Vv*NIPs expression is much lower than the observed in PIPs and TIPs (125% maximum) expressing strains [15]. *Vv*TnNIP1;1 showed significantly higher osmotic water and glycerol channel activity among all our studied grapevine NIPs. The phylogenic analysis of NIPs from various plant sources demonstrated an unambiguous clustering of *Vv*TnNIP1;1 in the NIP-I group, along with six *Arabidopsis* NIPs and the archetype *Gm*Nod26 (Figure 1). The functional characteristic of members of this group is comparable because they have been elucidated as permeable to both water and glycerol [5], which is in accordance with our findings. The ar/R filter of this NIP group is composed of hydrophilic amino acid residues, which tends to form a smaller pore (diameter ~2.8 Å) [12]. On the other hand, *Vv*TnNIP6;1 expression seems to enhance glycerol permeability across the membrane, but it was impermeable to water. *Vv*TnNIP6;1 grouped in the NIPII cluster (Figure 1), and the functional characterization of this group showed substantial variation in the ar/R region. It has hydrophobic amino acid residues and form a wider pore (diameter ~3.4 Å) [11]. The distinct size and hydrophobicity/philicity of the residues suggest that the members of this group have divergent substrate specificity. Similar to our result, *At*NIP6;1, a representative of this subgroup, showed glycerol permeability like NIP-I group but was impermeable to water despite presenting a wider aperture [20]. Different gating mechanisms [24] or the inability to organize the water molecules in the pore [25] were speculated as the possible reason for the incapability to transport water through group II members [5].

The physiological importance of glycerol in plants is still in question since no apparent role in osmoregulation was found in opposition to the aquaglyceroporin (Fps1) channel in yeast [26]. However, the role of glycerol as a carbon source in plants has been suggested [27], and the exogenous glycerol was demonstrated to affect the plant and root growth [28]. In plant–fungi symbiosis, *Gm*Nod26 is potentially significant to permeate glycerol along with NH_3_ during the process of nitrogen fixation [5,29].

Interestingly, each version of grapevine NIP6;1 (*Vv*TnNIP6;1 and *Vv*TnNIP6;1M) behaved differently for the water and glycerol transport activity. Expression of the mutated protein with longer C-terminal appeared to have higher permeability not only for glycerol but also for water (Figure 3 and Figure 4). Studies suggest that besides ar/R composition, precise amino acid stretch (108 amino acids) between the NPA motifs is also crucial for substrate selectivity and permeability [30,31]. To observe this requirement in grapevine NIPs for water and glycerol transport, obtained sequences were aligned with the representative NIPs sequences of their group. For instance, *Vv*TnNIP1;1 (NIP-I group, permeable to water and glycerol) was compared with *Gm*Nod26 and LMP2 sequences [23], and *Vv*TnNIP6;1 (NIP-II group, permeable to glycerol) was compared with *At*NIP6;1 sequence [20]. It was observed that the analyzed sequences do not have precise 108 amino acid stretch between NPA domains. While the amino acid stretches between NPA domains were consistent (109 amino acids) in the NIP-I group, it was different in the NIP-II group. *Vv*TnNIP6;1 has 110 amino acids, whereas *At*NIP6;1 has 108 amino acid distance between NPA domains. This finding suggests that for water transport, a precise length between NPA domains appeared to be important, whereas for glycerol transport, other factors are involved. 

The C-terminal extension of *At*NIP7;1 showed that this site could be phosphorylated in vitro [5]. The C-terminal has been shown to have multiple adjacent phosphorylation sites, which are considered crucial not only for aquaporin gating but also for phosphorylation-mediated membrane trafficking of the channel [32,33,34,35,36]. The well-studied phosphorylation site at Ser^262^ of *Gm*Nod26 [36] is conserved in grapevine *Vv*NIP1;1 and *Vv*NIP6;1 sequence. Six additional putative phosphorylation sites (Ser^314^, Ser^316^, Ser^317^, Thr^328^, Ser^342^, Ser^350^) were also predicted at the longer C-terminal of *Vv*TnNIP6;1M (Figure S2), which may result in enhanced channel activity by better gating and associated improved targeting of the channels to the plasma membrane. However, to ascertain this possibility, detailed in vivo/vitro phosphorylation studies in C-terminal variants of NIP homologs is a prerequisite. In addition to the possible gating regulation, this result also offers the possibility of physiological differences of the same aquaporin homolog in different varieties, which has been suggested previously in grapevine [37]. 

### 2.3. pH-Dependent Gating of Grapevine NIPs

The pH-dependent gating of grapevine NIPs, which were functional for water and glycerol (*Vv*TnNIP1;1 and *Vv*TnNIP6;1M) transport, was evaluated by measuring their permeabilities at different pH_out_ and pH_in_ conditions. At pH_out_ 6.8, the internal pH remained unaltered, whereas, at pH_out_ 5.0, pH_in_ dropped to 6.1. The addition of benzoic acid (4 mM) at pH_out_ 5.0 reduced the cytosolic pH to 4.8 while keeping the pH_out_ unaffected [18].

**Figure 6:**
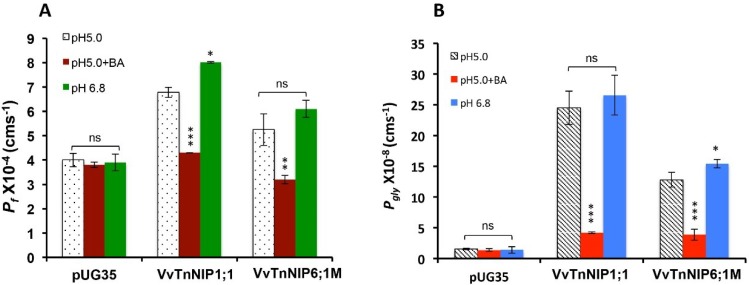
pH-dependent (**A**) water and (**B**) glycerol permeabilities of grapevine NIPs. Permeability coefficients were measured at extracellular pH 5.0 (pH_in_ 6.1), pH 5.0 + 4.0mM benzoic acid (BA) (pH_in_ 4.8), pH 6.8 (pH_in_ 6.8). Both functional NIPs (*Vv*TnNIP1;1 and *Vv*TnNIP6;1M) showed significantly reduced permeability for water and glycerol due to cytosolic acidification (pH_in_ 4.8). Whereas, yeast cells transformed with empty plasmid pUG35 was unaffected at all tested pH. Data is represented in the mean ± SD of three independent experiments with five traces. Statistically significant differences are shown as an asterisk, calculated by *t*-test (* *p* < 0.05, ** *p* < 0.01, and *** *p* < 0.001).

Dropping the pH_in_ from 6.8 to 6.1, slightly reduced the overall water as well as glycerol permeabilities of both *Vv*TnNIP1;1 and *Vv*TnNIP6;1M transformants (Figure 6). A sharp decline in water (4.3 ± 0.005 × 10^−4^ cm s^−1^) and glycerol (4.2 ± 0.15 × 10^−8^ cm s^−1^) permeabilities of *Vv*TnNIP1;1 was observed due to internal acidification by benzoic acid. Similarly, a significant lower channel activity for water (3.2 ± 0.18 × 10^−4^ cm s^−1^) and glycerol (3.87 ± 0.9 × 10^−8^ cm s^−1^) was noticed in *Vv*TnNIP6;1M strain due to lower internal pH (Figure 6). The estimated activation energies for water and glycerol transport in each condition were correspondingly in accordance with their permeabilities (Table 1).

Numerous studies demonstrated that the regulation of water permeability through PIPs and TIPs is mediated by cytosolic pH-dependent gating of the channels [18,38,39]. The structural model of PIP2 members suggests that a His residue in the cytoplasmic loop D acts as a pH sensor, which can be protonated by cytosolic acidification during waterlogging, resulting in stabilization of the closed conformation of aquaporins [38,40]. The internal pH-sensitivity of *Vv*TnNIP1;1 and *Vv*TnNIP6;1M channels, led us to explore the conserved His residue in NIPs sequences. Surprisingly, all NIPs lack any His residue in loop D, implying that this residue is not the sole pH-sensory site. In fact, its mutation merely reduced the pH sensitivity of the channel to some extent, while the double mutation of the His along with other acidic amino acids (R194 and D195), completely abolished the cytosolic pH-sensitivity [38]. This finding opens up the possibility of participation of other cytosolic acidic amino acids in the intracellular pH-sensitivity of grapevine NIPs. Sequence analysis identified the constitutive presence of acidic amino acids, Asp (D^186^ in *Vv*TnNIP1;1, D^219^ in *Vv*TnNIP6;1) and Arg (R^186^ in *Vv*TnNIP1;1 and R^221^ in *Vv*TnNIP6;1), in loop D of all NIPs (Figure S3). Additionally, the existence of many pH-sensitive aquaporins (including mammalian aquaporins) without His residue in loop D [38], may imply other candidate amino acids, with high pKa values and with the ability to form hydrogen bonding, as pH-sensor [41]. 

### 2.4. Growth Assays of Transformed Yeast Strains for Screening the Substrate Selectivity Profile of Grapevine NIPs

Yeast strains expressing grapevine NIPs were plated on different concentrations of various substrates. Their tolerance/sensitivity towards externally applied substrates may be an indication of their putative involvement in the transport of test substrates.

#### 2.4.1. Metalloids

In the last decade, the understanding of metalloid transport in plants received more attention and progression [42]. The molecular and physiological studies suggest that NIPs exhibit varying degrees of pore selectivity for substrates other than water and that they are essential not only for beneficial metalloid transport but also for translocation and extrusion of toxic substrates in the plant [6]. The suggested role of NIPs as metalloido-porins [6] provided a notion to perceive their role in grapevine for metalloids transport.

##### Arsenium

**Figure 7:**
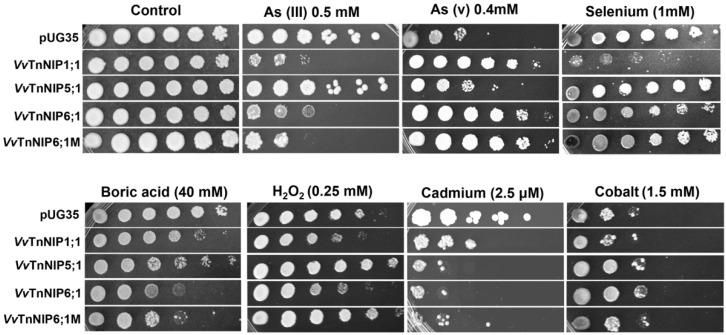
Yeast strains expressing grapevine NIPs exhibited sensitivity toward metalloids (As, Se, and B), H_2_O_2_, and heavy metals (Cd and Co). *S. cerevisiae aqy-null* strain was transformed either with empty plasmid pUG35 or with the plasmid containing *VvTnNIP1;1*, *VvTnNIP5;1*, *VvTnNIP6;1*, and *VvTnNIP6;1M*, were spotted in 10-fold dilution on plates containing indicated concentration of test substrates. Minimal media plate without additional substrate is considered as control media. Growth was recorded after one week at 28 °C. Photographs shown are representative of at least two independent experiments having two replicate plates showing consistent results.

**Figure 8:**
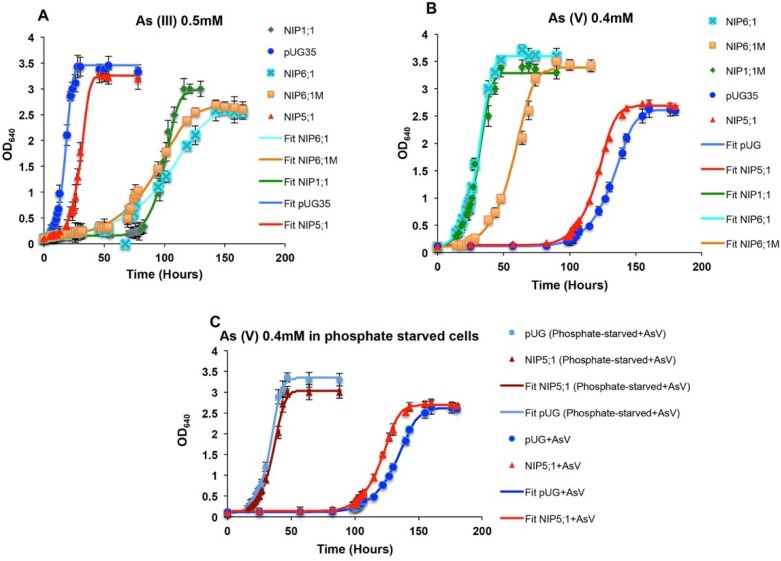
Growth assays of *S. cerevisiae* strains in arsenium. Yeast cells expressing empty plasmid pUG35 or grapevine NIPs were washed and inoculated (A_260_ = 0.1) in the minimal media containing (**A**) 0.5 mM arsenite (As III) and (**B**) 0.4 mM arsenate (As V). (**C**) In another set of experiments, yeast strains (control and *Vv*TnNIP5;1), which did not show involvement in As (III) flux and hence were sensitive to As (V), were grown in low-phosphate media. Thereafter, the phosphate-starved cells were subjected to grow in the presence of As (V), under phosphate-replete condition, showing abolished arsenate toxicity in these strains. Obtained data points of the curve were fitted to the ComBase tool DMFit (online) [30] and growth parameters like specific growth rate (h^−1^) and final biomass of the fits were estimated (Table S2). Data is represented in the mean ± SD of at least two independent experiments with three replicates.

Grapevine NIP aquaporins were tested for their involvement in the transport of arsenium. Growth assays of NIPs expressing yeast strains were performed in the presence of arsenite (As III) and arsenate (As V) (Figure 7 and Figure 8, Table S2). As (III) triggers toxicity by interfering with the overall protein functions leading to growth impairment. In plants, As (III) enters the cells through NIPs/PIPs, whereas in yeast, it is transported through Fps1 [43]. Growth of *Vv*TnNIP1;1 and *Vv*TnNIP6;1 expressing strains was repressed in the presence of 0.5 mM As (III) as compared to control strain, suggesting the involvement of these NIPs in As (III) influx (Figure 7 and Figure 8A, Table S2). In contrast, the same strains showed tolerance towards externally supplied 0.4 mM As (V) (Figure 7 and Figure 8B, Table S2). As (V) is a non-reactive phosphate analogue. In higher plants, it is taken up through phosphate transporters (PHT1) and rapidly reduced to As (III) by arsenate reductase (ACR2) for subsequent sequestration in vacuole by ABC transporters [43]. Additionally, cellular extrusion of As (III) can occur by NIPs/PIPs down the concentration gradient to the external medium. Our data demonstrates that expression of specific NIPs (*Vv*TnNIP1;1 and *Vv*TnNIP6;1) sensitize the yeast cells to As (III), whereas expression of the same NIPs improved growth on As (V) containing media, probably due to increased efflux of As (III), which was generated from As (V) reduction. On the other hand, yeast cells transformed with *Vv*TnNIP5;1 could not show sensitivity to As (III) but was sensitive to As (V), suggesting that *Vv*TnNIP5;1 is not involved in transmembrane As (III) flux (Figure 7 and Figure 8, Table S2). The As (V)-sensitivity of control and *Vv*TnNIP5;1 expressing cells was alleviated when phosphate-starved cells were grown in the presence of As (V) and phosphate together (Figure 8C, Table S2). The result supports the notion that both substrates are transported through the same transmembrane route, and absorption of phosphate is preferred over arsenate [44].

Taken together, the growth assays in As (III) and As (V) suggest that both *Vv*TnNIP1;1 and *Vv*TnNIP6;1 play a possible role in influx/efflux of As (III), behaving as bidirectional channels for As (III). Numerous studies of bidirectional arsenic channels have also been established within the NIP subfamily of *Arabidopsis*, rice, and lotus [19,45,46,47]. In addition to uptake, a recent study suggests that NIPs are also involved in As (III) translocation and distribution from roots to the distal parts of the plant [48].

##### Selenium (Se)

**Figure 9:**
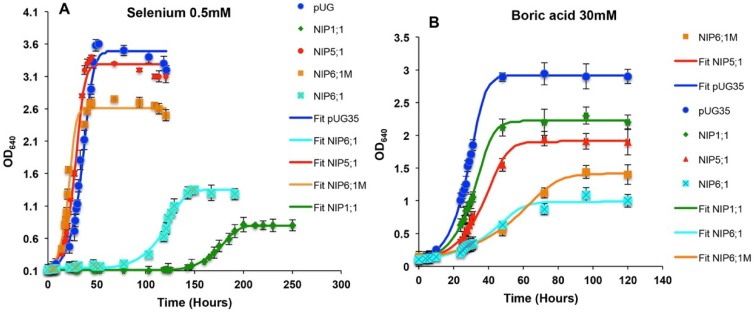
Growth assays of *S. cerevisiae* strains in the presence of (**A**) selenium (0.5 mM) and (**B**) Boron (30 mM). Yeast cells expressing empty plasmid pUG35 or grapevine NIPs were washed and inoculated (A_260_ = 0.1) in the minimal media containing test substrates. Obtained data points of the curve were fitted to the ComBase tool DMFit (online) [30] and growth parameters like lag phase (h), specific growth rate (h^−1^), and final biomass of the fits were estimated (Table S2). Data is represented in the mean ± SD of at least two independent experiments with three replicates.

Yeast strains expressing grapevine NIPs showed a varying degree of sensitivity towards selenium. *Vv*TnNIP5;1 and *Vv*TnNIP6;1M expressing strains did not exhibit any phenotypic growth behavior, whereas the strains expressing *Vv*TnNIP1;1 and *Vv*TnNIP6;1 showed decreased growth in the presence of selenium both on solid and in liquid culture (Figure 7 and Figure 9A). *Vv*TnNIP1;1 expressing strain had the lowest specific growth rate and final biomass, whereas *Vv*TnNIP6;1 also grew at a slower rate but acquired higher biomass (Table S2). These results suggest that these two NIPs in grapevine may have a possible contribution to the transport of selenium.

Selenium exists in soil mainly as selenate (VI) and selenite (IV). Their prevalence depends on the pH and redox potential of the rhizosphere [6]. Numerous evidence has shown that selenate (VI) is majorly transported via sulfate transporters due to their structural and chemical similarities [49]. On the contrary, little is known about the selenite transport mechanism in plants. The uptake kinetics studies of selenite in maize roots suggest that it can be taken up through MIPs [50]. The rice *Os*NIP2;1 (NIP-III group with largest pore size) was the first identified selenium transporter [51], which was previously reported as the first silicon transporter (Lsi1) in plants [52]. Since it is considered that plants do not require selenium for growth, its transport is assumed to be a non-physiological secondary activity of the silicic acid permeable NIPs [6]. On the other hand, plants benefit from the accumulated Se. It promotes plant growth, yield, and resistance to abiotic stress and pathogens [53]. Our results indicate that grapevine NIPs of other groups like *Vv*TnNIP1;1 (NIP-I group) and *Vv*TnNIP6;1 (NIP-II group) are putatively permeable to selenite, suggesting that their permeability is not restricted to the NIP-III group.

##### Boron (B)

All grapevine NIPs expressing strains showed increased growth susceptibility to boric acid on solid as well as in liquid media (Figure 7 and Figure 9B, Table S2). However, the control strain grew well in the presence of boric acid. Cells expressing *Vv*TnNIP6;1 (both native and mutated protein) exhibited the highest sensitivity, while strain expressing the *Vv*TnNIP5;1 was less sensitive to boric acid (Figure 7 and Figure 9). 

Boron is an essential micronutrient but is toxic at higher concentrations [54], and its imbalances affect both yield and quality of the plants [55]. Boron uptake is a combination of passive diffusion and channel-mediated transport [56], especially under limited boron conditions [57]. An interesting network of boron uptake and translocation in plants was suggested [58]. In *Arabidopsis*, the uptake of boron from soil to root cells is mediated by *At*NIP5;1 channel, and it is loaded into the xylem by BOR1, a borate exporter. After that, a water-impermeable channel *At*NIP6;1 facilitates the xylem-phloem transfer of boric acid at the nodal regions in the aerial part of the plants. Several studies have shown that NIPII group members are crucial for boron transport [59,60]. Accordingly, our results also indicate that among all studied NIPs, the NIP-II water-impermeable *Vv*TnNIP6;1, showed the most prominent putative role in boron transport (Figure 7 and Figure 9B, Table S2).

#### 2.4.2. H_2_O_2_

The expression of *Vv*TnNIP1;1 and *Vv*TnNIP6;1 caused slight sensitivity to the yeast cells when grown in the presence of 0.25 mM H_2_O_2_, whereas general toxicity was observed at higher concentrations (Figure 7, Table S2). H_2_O_2_ is a dual nature molecule. It is a threatening reactive oxygen species (ROS) triggering cell death as well as a signal molecule to activate Ca^+2^ for root hair growth and stomatal movement [61]. Previous studies demonstrated that unlike the PIPs and TIPs [62,63,64], the H_2_O_2_ permeability through NIPs has barely been observed [62,65]. The lower H_2_O_2_ conductance through NIPs may be linked with their lower water permeability because water and hydrogen peroxide possess similar molecular characteristics and permeabilities [66].

#### 2.4.3. Heavy Metals

In order to investigate the putative transport of heavy metals by grapevine NIPs, we performed growth assays in the presence of positively charged heavy metals like Cd, Cu, and Co. Growth of the yeast cells was affected only in the presence of Cd, whereas slight sensitivity of *Vv*TnNIP1;1 strain to Co was also observed (Figure 7). Expression of *Vv*TnNIP5;1 and both homologs of NIP6;1 (*Vv*TnNIP6;1 and *Vv*TnNIP6;1M) caused more sensitivity as compared to *Vv*TnNIP1;1 expression (Figure 7, Table S2) in the presence of Cd. Our results indicate that grapevine NIPs may facilitate the transport of positively charged heavy metals. Similarly, grapevine XIP aquaporin was demonstrated to transport Cu and Ni [16]. In addition to the conductance through aquaporins, heavy metals have been shown to impair the overall aquaporin activity by modulating the gating of the channel [67,68]. Similar to mercury (Hg), Cd, and other heavy metals like lead (Pb) and zinc (Zn) can also block the aquaporins, consequently disturbing the plant water balance [69]. The impairment of water conductance is considered as a first feedback response to heavy metal toxicity [68], indicating the significant role of aquaporins during heavy metal stress in plants.

## 3. Materials and Methods

### 3.1. Yeast Strain, Vector, and Growth Conditions

The centromeric plasmid pUG35 conferring C-terminal GFP tagging, MET25 promoter, and CYC1-T terminator [70] was used for the cloning of putative NIPs aquaporins from *V. vinifera* cv. Touriga Nacional. To express these aquaporins, *S. cerevisiae* YSH1172 strain (10560-6B *MATa leu2::hisG trp1::hisG his3::hisG ura3-52 aqy1::KanMX4 aqy2::HIS3*) in which native aquaporins (*AQY1* and *AQY2*) were deleted, from now on designated as *aqy-null*, was used. Yeast transformants were grown and maintained in Yeast Nitrogen Base (YNB) medium without amino acids (DIFCO) with 2% (w/v) glucose supplemented with the adequate requirements for prototrophic growth [71].

### 3.2. Sequence Analysis, Cloning, and Expression of Grapevine NIPs in S. cerevisiae

The full-length putative grapevine NIPs ORFs were amplified from cDNA of *V. vinifera* cv. Touriga Nacional (kindly provided by Dr. Luísa Carvalho, ISA-ULisboa) and cloned before GFP sequence in the pUG35 vector, by following our previously ascribed protocols [15,18]. The primers used in this study and the NCBI accession numbers of the cloned NIPs are listed in Table S1. The plasmid constructs were expressed in *aqy-null S. cerevisiae*, as mentioned in our previous report [15]. The membrane localization of the expressed GFP-tagged grapevine NIPs in *S. cerevisiae* was confirmed under Leitz Wetzlar Germany 513558 epifluorescence microscope equipped with a Leitz Wetzlar Germany Type 307-148002 514687 mercury bulb and BP 340–380; BP 450–490 (for GFP visualizing); BP 515–560 filter sets. Images were obtained with a digital camera Axiocam Zeiss using AxioVision Rel. 4.8.2 Software [15].

Topology and hydrophobicity of deduced amino acid sequences of grapevine NIPs were predicted by using at least three ExPASy tools, e.g., TMHMM [72], HMMTOP [73], and TMPred [74]. The sequences obtained from the present study were aligned with the sequences of *V. vinifera* cv. Pinot Noir [13]. Conserved amino acid residues at NPS/V and ar/R constrictions were determined from the ClustalX [75] and BioEdit [76] alignments with other NIPs homologs of various plant species. The obtained alignment was used to construct the phylogenetic tree of the NIP group by MEGA7.0 software using neighbor-joining method [22]. The putative phosphorylation sites of NIPs were predicted by NetPhos 3.1 server [77].

### 3.3. Water and Glycerol Transport Assays by Stopped-Flow Spectroscopy

Membrane water and glycerol permeabilities of the yeast cells expressing grapevine NIPs were measured by stopped-flow fluorescence spectroscopy (HI-TECH Scientific PQ/SF-53), as previously described by [15,78]. For measuring the osmotic water permeability (*P_f_*), 100 µL of yeast cells equilibrated in 1.4 M sorbitol and loaded with fluorophore precursor 5-(and-6)-carboxyfluorescein diacetate (CFDA), was mixed with an equal volume of hyperosmotic solution 2.1 M sorbitol creating an inwardly directed sorbitol gradient and leading to water efflux and cell shrinkage. Cell shrinkage induces quenching of the fluorescent dye with consequent decrease of the signal output. The kinetics of cell shrinkage was followed until a stable fluorescent signal was attained. Signals were fitted to a single exponential, from which the rate constant (k) was calculated. *P_f_* was estimated by *P_f_* = *k (V_o_/A)(1/V_w_(osm_out_)_∞_)*, where *V_w_* is the molar volume of water, *V_o_/A* is the initial cell volume to area ratio, and *(osm_out_)_∞_* is the final medium osmolarity after the applied osmotic gradient.

For glycerol permeability (*P_gly_*), equal volumes of prepared yeast cells and 2.1 M glycerol solution were mixed creating an inwardly directed glycerol gradient, which leads to rapid cell shrinkage caused by water efflux, followed by cell re-swelling due to glycerol influx and consequent water influx. The rate of re-swelling due to glycerol influx was measured as the slope of a linear regression fit. *Pgly* was estimated from *P_gly_* = *m(V_o_/A)*, where *m* is the linear slope fitted to the signal of glycerol influx. Experiments were performed at 23 °C for water and glycerol permeabilities.

For activation energy (*E_a_*), cells were subjected to osmotic shocks at various temperatures, ranging from 10 °C to 35 °C. Activation energies (*E_a_*) were evaluated from the slope of an Arrhenius plot (ln*P_f_* or ln*P_gly_* as a function of 1/T). 

For inhibition of channel activity, cells were incubated with 0.5 mM HgCl_2_ for 15 min at room temperature before stopped-flow experiments. To demonstrate the reversal of HgCl_2_ mediated inhibition, cells were further incubated with 3 mM β-mercaptoethanol for 10 min before the osmotic shock.

### 3.4. Effect of pH on Gating of Grapevine NIPs

To evaluate the pH-dependent gating of NIPs, yeast cells expressing grapevine NIPs were washed and incubated under different pH conditions (external pH 5.0, external pH 5.0 + 4.0 mM benzoic acid, and external pH 6.8) in 1.4 M isotonic sorbitol solution for 90 min on ice before permeability assays as described previously [18]. 

The intracellular pH (pH_in_) of yeast transformants was determined from the relative distribution of labeled ^14^[C]-propionic acid [79] using the same conditions described above. 

### 3.5. Growth Assays for Screening of Substrate Selectivity Profile of Grapevine NIPs

#### 3.5.1. Substrates Other Than Water and Glycerol

To determine the putative involvement of grapevine NIPs in transport of non-aqua substrates, a tolerance/sensitivity test of the yeast strains expressing *Vv*TnNIP1;1, *Vv*TnNIP5;1, and *Vv*TnNIP6;1 was performed by growth assays in the presence of selected substrates. The tested putative substrates were arsenate (As V) (as sodium arsenate 0.2, 0.4, 0.8 mM), arsenite (As III) (as sodium arsenite 0.5, 1.0, 1.5 mM), boron (B) (as boric acid, 20, 40, and 60 mM), cadmium (Cd) (as cadmium sulphate (1.0, 2.5, and 5.0 μM), copper (Cu) (as copper sulphate 0.25, 0.50, 1.0 mM), cobalt (Co) (as cobalt chloride 1, 2, and 4 mM), hydrogen peroxide (H_2_O_2_) (0.25, 0.5, 0.75, 1.0, 1.5, and 2.0 mM), nickel (Ni) (as nickel chloride 2.5, 5, and 10 mM), and selenium (Se) (as sodium biselenite 0.1, 0.5, 1 mM). Stock solutions of test substrates (50 mM) were prepared and their pH (5.0) was adjusted with Tis-base/HCl, prior to adding to the media. In the case of boric acid 200 mM stock solution was prepared.

#### 3.5.2. Low-Phosphate Media

Phosphate-starved cells were grown in the presence of As (V) and phosphate together to test the effect of phosphate on As (V) transport. A low phosphate medium was prepared by following the method described by [80]. Inorganic phosphate was precipitated (as MgNH_4-_PO_4_) from 1% (*w/v*) bacto-yeast extract and 2% (*w/v*) bacto-peptone by the addition of 10 mL MgSO_4_ (1 M), and 10 mL concentrated aqueous ammonia, followed by incubation for 30 min at room temperature. The precipitates were filtered out through Whatman No. 1 filter paper, and pH (5.8) of the media was adjusted with HCl and autoclaved. After cooling the media, sterile glucose was added to the final concentration of 2% (*w/v*).

#### 3.5.3. Drop-Test and Growth Assay

Drop tests were performed for tolerance/sensitivity assessment. Actively growing yeast strains were harvested at the early exponential phase (OD_640 nm_ ≈ 0.6–0.8). After centrifugation, the cells were re-suspended in sterile distilled water at OD_640 nm_ ≈ 10, being further serially diluted at 10-fold, and 3 µL was spotted with replica platter for 96-well plates device on plates containing YNB solid medium with test substrates. Yeast strain with an empty vector (pUG35) was considered as the negative control. Differences in growth were scored after 1–2 weeks of incubation at 28 °C.

To confirm the tolerance/sensitivity pattern obtained from the above-mentioned drop-test, growth assays were performed in liquid media with test substrates. Yeast strains were grown to exponential phase and then diluted to OD_640 nm_ ≈ 0.1 in YNB liquid medium with or without the test substrate. Growth was recorded by periodically measuring the OD_640 nm_. Obtained data points of the curve were fitted to the ComBase tool DMFit (online) [81], and growth parameters like lag phase (h), specific growth rate (h^−1^), and final biomass of the fits were estimated.

### 3.6. Statistical Analysis

All the data were collected from at least three independent experiments with consistent results. In the case of stopped-flow experiments, typically five hyperosmotic shocks at each temperature and at least ten shocks at 23 °C were run and analyzed in each experiment. The data were analyzed by Student’s *t*-test, and asterisks indicate statistically significant differences, where *p* < 0.05 (marked as *), *p* < 0.01 (marked as **), and *p* < 0.001 (marked as ***). Data are presented as mean ± standard deviation (SD).

## 4. Conclusions

The combined sequence and phylogenetic analysis, transport assays, and phenotypic growth data presented above demonstrate the ability of NIPs to transport water and glycerol, and support evidence that they are involved in transmembrane flux of As, B, Se, and Cd. These findings are in agreement with the expected group-specific substrate profile, but metalloid selection did not strictly follow the assumed substrate range of the particular group. We found that *Vv*TnNIP1;1 (NIP-I group) was highly permeable to glycerol and water, and was putatively involved in the conductance of As (III) Se, B, and H_2_O_2_. On the other hand, *Vv*TnNIP6;1 (NIP-II group) was moderately permeable to glycerol, and putatively facilitated As (III), Cd, B, and H_2_O_2_ transport. Whereas, *Vv*TnNIP5;1, another member of group II, could not facilitate the transport of any substrate except Cd. The finding suggests that substrate selection of the specific NIP does not merely depend on their group. Other structural features, along with the specific constriction region of each group, appear to play a role in substrate selection. The substrate-profile of each NIP homolog may vary in different plants and species, depending on the prevalence of metalloids and their environmental conditions. Despite the overlapping substrate profile of NIPs and the concomitant presence of toxic as well as beneficial metalloids in the soil, the efficient and specific substrate uptake through NIPs confers a fine-tuned mechanism of solute selection. Interestingly, it was demonstrated that NIPs could efficiently discriminate between the structurally similar As (III), Sb (III), and glycerol [19]. The elucidation of NIPs functionality in this regard will contribute to our further understanding of water and solute relations in the whole plant.

Additionally, improved channel activity of mutated version of *Vv*TnNIP6;1M (with longer C-terminal), supports the significance of the C-terminal length for NIPs functionality in yeast, inferring the possible gating regulation through phosphorylation and associated membrane trafficking of NIPs. Further phosphorylation related investigation is a prerequisite to establishing this suggestion. This finding also implies the distinct physiological significance of the same aquaporin homolog in different varieties.

Further studies on mutant NIP aquaporins (at key residues of ar/R constriction) expressed in single deletion yeast strains, defective for the test substrate transporter, can be performed to establish the link between structural-functional aspects of NIPs in a cleaner background. The findings will offer a comprehensive knowledge of the physiological importance of NIPs for metalloid transport and phytoremediation of toxic metals in plants, particularly in grapevine. 

## Figures and Tables

**Table 1:** Activation energy (*E_a_*) for water and glycerol transport at different intracellular pH.

Strains	Activation Energy for Water Transport *E_a_* (kcal mol^−1^)			Activation Energy for Glycerol Transport *E_a_* (kcal mol^−1^)		
	pH_out_ 5.0	pH_out_ 5.0 + BA ^1^	pH_out_ 6.8	pH_out_ 5.0	pH_out_ 5.0 + BA ^1^	pH_out_ 6.8
	(pH_in_ 6.1)	(pH_in_ 4.8)	(pH_in_ 6.8)	(pH_in_ 6.1)	(pH_in_ 4.8)	(pH_in_ 6.8)
pUG35	14.05 ± 0.01	13.80 ± 0.2	13.67 ± 0.4	24.30 ± 1.2	25.10 ± 0.8	24.20 ± 0.98
*Vv*TnNIP1;1	9.80 ± 0.15	12.78 ± 0.16	9.53 ± 0.1	6.93 ± 0.22	11.69 ± 0.34	9.11 ± 0.023
*Vv*TnNIP5;1	14.60 ± 0.8	nd	nd	24.50 ± 1.1	nd	nd
*Vv*TnNIP6;1	13.54 ± 0.2	nd	nd	12.55 ± 0.8	nd	nd
*Vv*TnNIP6;1M	11.3 ± 0.4	14.34 ± 0.26	11.93 ± 0.93	8.6 ± 0.5	12.11 ± 1.9	8.54 ± 0.4

^1^ BA: 4.0 mM benzoic acid.

## Supplementary Materials

The following supplementary materials can be found at https://www.mdpi.com/1422-0067/21/2/663/s1. Figure S1: Alignment of grapevine *NIP6;1* of Pinot Noir and Touriga Nacional cultivars, showing the four base pair insertion in *VvTnNIP6;1* resulting in a shorter protein; Figure S2: The alignment is showing the different C-terminal length of grapevine NIP6;1 of Pinot Noir (*Vv*PnNIP6;1) and Touriga Nacional (*Vv*TnNIP6;1) varieties. C-terminal extension in mutated homolog (*Vv*TnNIP6;1M) shows similarity with NIP6;1 homolog of Pinot Noir cultivar. The alignment also exhibits the putative phosphorylation sites (highlighted in red) at C-terminal of functional grapevine NIPs obtained in the present study, and *Gm*Nod26 from *Glycine max*. Predicted phosphorylation sites at Ser residue at C-terminal are highlighted. The alignment shows six additional tentative phosphorylation sites in longer C-terminal homologs (*Vv*PnNIP6;1 and *Vv*TnNIP6;1M); Figure S3: Putative pH-sensitive sites at the cytoplasmic loop D for NIPs gating. Sequences obtained in the present study are marked with an arrow. The alignment is showing the absence of highly conserved His residue for pH-sensitivity in loop D of all aligned NIPs sequences. Whereas, the consecutive presence of acidic amino acids (Asp and Arg) was observed, which possibly present the internal pH-sensors at the cytoplasmic loop; Table S1: Primer sequences used in this study; Table S2: Growth parameters of *S. cerevisiae* strains expressing grapevine NIPs in minimal media containing test substrates.
