# Supplementary material for: Molecular and Functional Characterization of Grapevine NIPs through Heterologous Expression in aqy-Null Saccharomyces cerevisiae
Source: Int J Mol Sci. 2020 Jan 19;21(2):663. doi: 10.3390/ijms21020663 (PMC7013980; doi:10.3390/ijms21020663)
Supplement: Supplementary file 1 [file ijms-21-00663-s001.zip › ijms-664942-SI-to conversion/Supplementary Tables S1-S2.pdf]

Supplementary Tables:

Table S1. Primer sequences used in this study.

Genes	NCBI Accession Number	Primer Name	Primer Sequence (5′–3′)
NIP1;1	MN723560	N11_F	GCGAGCTCTAGATAGCTAGAACTGATGGGCGAGATATCAG
		N11_R	ATACGCGTCGACAGACCCATTACGGGACGATTTGC
NIP5;1	MN723561	N51_F	GCGAGCTCTAGATTCTCAATGGCGGAAGCAGAAACTGG
		N51_R	ATACGCGTCGACGCGACGGAAGCTCCTGACC
NIP6;1	MN723562	N61_F	GCGAGCTCTAGAATGGACACAGACCATGATGTTCCATCAG
		N61_R	ATACGCGTCGACTGTATTGTATGTACTGGCCGGATCTCGATC
NIP6;1M	MN723563	N61_FM	AAGTCCATGACGGTGCAGAGCGAAGGTGACCAGTCAATG
		N61_RM	CATTGACTGGTCACCTTCGCTCTGCACCGTCATGGACTT

*Restriction enzyme sequences are underlined.

Table S2. Growth parameters of *S. cerevisiae* strains expressing grapevine NIPs on minimal media containing atypical substrates.

Substrates	Growth parameters	Strains				
		pUG35	NIP1;1	NIP5;1	NIP6;1	NIP6;1M
Control	Specific growth rate (hour^{-1})	0.2±0.005	0.195±0.008	0.193±0.003	0.2±0.006	0.189±0.007
	Final biomass	4.1±0.21	4.7±1.05	4.25±1.10	4.1±0.09	4.2±0.09
Selenium (0.5mM)	Specific growth rate (hour^{-1})	0.089±0.005	0.036±0.003	0.102±0.004	0.036±0.005	0.13±0.007
	Final biomass	3.48±1.05	0.80±1.03	3.24±1.03	1.35±1.08	2.62±1.04
As (III) (0.5mM)	Specific growth rate (hour^{-1})	0.17±0.001	0.08±0.002	0.13±0.002	0.027±0.003	0.032±0.004
	Final biomass	3.46±1.0	2.98±1.20	3.26±1.60	2.64±1.08	2.68±1.04
As (V) (0.4mM)	Specific growth rate (hour^{-1})	0.056±0.002	0.106±0.005	0.068±0.003	0.101±0.004	0.067±0.003
	Final biomass	2.65±1.03	3.30±1.03	2.69±1.04	3.6±1.03	3.38±1.05
As (V) (0.4mM) (Phosphate-starved cells)	Specific growth rate (hour^{-1})	0.12±0.002	---	0.107±0.002	---	---
	Final biomass	3.35±1.02	---	3.04±1.02	---	---
Boric acid (30mM)	Specific growth rate (hour^{-1})	0.102±0.001	0.085±0.001	0.068±0.006	0.044±0.004	0.034±0.003
	Final biomass	2.91±1.02	2.22±1.01	1.91±1.06	1.02±1.05	1.42±1.06
H_2O_2 (0.25mM)	Specific growth rate (hour^{-1})	0.18±0.017	0.131±0.010	0.183±0.006	0.156±0.010	0.203±0.008
	Final biomass	4.06±1.10	3.63±0.09	3.92±1.03	3.56±1.02	4.0±1.10
Cadmium (2.0µM)	Specific growth rate (hour^{-1})	0.15±0.002	0.055±0.003	0.02±0.003	0.018±0.006	0.035±0.004
	Final biomass	3.06±1.10	2.1±1.05	1.0±0.01	0.85±0.01	0.98±0.02
