# Supplementary material for: Molecular and Functional Characterization of Grapevine NIPs through Heterologous Expression in aqy-Null Saccharomyces cerevisiae
Source: Int J Mol Sci. 2020 Jan 19;21(2):663. doi: 10.3390/ijms21020663 (PMC7013980; doi:10.3390/ijms21020663)
Supplement: Supplementary file 1 [file ijms-21-00663-s001.zip › ijms-664942-SI-to conversion/Supplementray Figure S1.pdf]

```
                              910       920       930       940       950
                     ....|....|....|....|....|....|....|....|....|....|..
VvPnNIP6;1           CTCAACAAGTCCATGACGGTGCAGAGC----GAAGGTGACCAGTCAATGTCA
VvTnNIP6;1           CTCAACAAGTCCATGACGGTGCAGAGCTTCAGAAGGTGACCAGTCAATGTCA
Clustal Consensus    ***************************    *********************
```

Figure S1: Alignment of grapevine *NIP6;1* of Pinot noir and Touriga nacional cultivar, showing the four base pair insertion in *VvTnNIP6;1* resulting in shorter protein.
