# Supplementary material for: Molecular and Functional Characterization of Grapevine NIPs through Heterologous Expression in aqy-Null Saccharomyces cerevisiae
Source: Int J Mol Sci. 2020 Jan 19;21(2):663. doi: 10.3390/ijms21020663 (PMC7013980; doi:10.3390/ijms21020663)
Supplement: Supplementary file 1 [file ijms-21-00663-s001.zip › ijms-664942-SI-to conversion/Supplementray Figure S2.pdf]

```
                             310       320       330       340       350
                    ....|....|....|....|....|....|....|....|....|....|....
GmNod26             KSASFLKGRA-------------ASK----------------------------
VvTnNIP1;1          KSGSFLKSKS-------------SRNGS--------------------------
VvPnNIP1;1          KSGSFLKSKS-------------SRNGS--------------------------
VvTnNIP6;1          LNKSMTVQS--------------FRR----------------------------
VvTnNIP6;1M         LNKSMTVQSEGDQSMSSVGLQHKFRNFTRKVRQFFEEIRNNSPMDRDPASTYNT
VvPnNIP6;1          LNKSMTVQSEGDQSMSSVGLQHKFRNFTRKVRQFFEEIRNNSPMDRDPASTYNT
Clustal Consensus    . *:                    .
```

Figure S2: The alignment is showing the different C-terminal length of grapevine NIP6;1 of Pinot noir (*Vv*PnNIP6;1) and Touriga nacional (*Vv*TnNIP6;1) variety. C-terminal extension in mutated homolog (*Vv*TnNIP6;1M) shows similarity with NIP6;1 homolog of Pinot noir cultivar. The alignment also exhibits the putative phosphorylation sites (highlighted in red) at C-terminal of functional grapevine NIPs obtained in the present study, and GmNod26 from *Glycine max*. Predicted phosphorylation sites at Ser residue at C-terminal are highlighted. The alignment shows six additional tentative phosphorylation sites in longer C-terminal homologs (*Vv*PnNIP6;1 and *Vv*TnNIP6;1M) .
