# Supplementary material for: Molecular and Functional Characterization of Grapevine NIPs through Heterologous Expression in aqy-Null Saccharomyces cerevisiae
Source: Int J Mol Sci. 2020 Jan 19;21(2):663. doi: 10.3390/ijms21020663 (PMC7013980; doi:10.3390/ijms21020663)
Supplement: Supplementary file 1 [file ijms-21-00663-s001.zip › ijms-664942-SI-to conversion/Supplementray Figure S3.pdf]

Loop D

AtNIP1;2	TDNRAIG	203
AtNIP4;1	TDNRAVG	187
AtNIP4;2	TDSRATG	187
GmNod26	TDNRAVG	182
MtNIP2	TDNRAIG	182
OsNIP1;1	TDNRAIG	192
OsNIP1;2	TDNRAIG	214
OsNIP1;3	TDNRAIG	198
OsNIP1;4	TDDQAVG	200
Ps Nod26	TDNRAIG	183
VuNIP1;1	TDNRAIG	183
VvPnNIP1;1	TDNRAIG	192
VvTnNIP1;1	TDNRAIG	192
VvPnNIP3;1	VVNKIYG	186
ZmNIP1;1	TDNRAIG	188
AnNIP1;1	TDTRAVG	217
AtNIP5;1	TDTRAVG	221
AtNIP6;1	TDTRAVG	223
AtNIP7;1	CDFVQLG	189
OsNIP3;2	TDPNAVK	238
OsNIP3;3	TDPNAVK	211
OsNIP4;1	TDGTAGK	200
VvPnNIP5;1	TDTRAVG	215
VvTnNIP5;1	TDTRAVG	215
VvPnNIP6;1	TDTRAVG	224
VvTnNIP6;1M	TDTRAVG	224
VvTnNIP6;1	TDTRAVG	224
LjNIP6;1	TDTRAVG	226
ZmNIP3;1	TDTRAVG	219
CpNIP2;1	TDTKAVG	191
CaNIP2;1	TDPKAIG	175
OsNIP2;1	TDTRAVG	192
OsNIP2;2	TDSRAVG	195
VvPnNIP2;1	TDTKAIG	191
VvTnNIP2;1	TDTKAIG	191
VvPnNIP7;1	SQPQSVS	205
ZmNIP2;1	TDTRAVG	190
ZmNIP2;2	TDSRAVG	195
ZmNIP2;3	TDSRAVG	198

Figure S3: Putative pH-sensitive sites at the cytoplasmic loop D for NIPs gating. Sequences obtained in the present study are marked with an arrow. The alignment is showing the absence of highly conserved His residue for pH-sensitivity in loop D of all aligned NIPs sequences. Whereas, the consecutive presence of acidic amino acids (Asp and Arg) was observed, which possibly present the internal pH-sensors at the cytoplasmic loop.
